# Supplementary material for: Clade 2.3.4.4b but not historical clade 1 HA replicating RNA vaccine protects against bovine H5N1 challenge in mice
Source: Nat Commun. 2025 Jan 14;16:655. doi: 10.1038/s41467-024-55546-7 (PMC11732985; doi:10.1038/s41467-024-55546-7)
Supplement: Supplementary file 1 — Supplementary Information [file 41467_2024_55546_MOESM1_ESM.pdf]

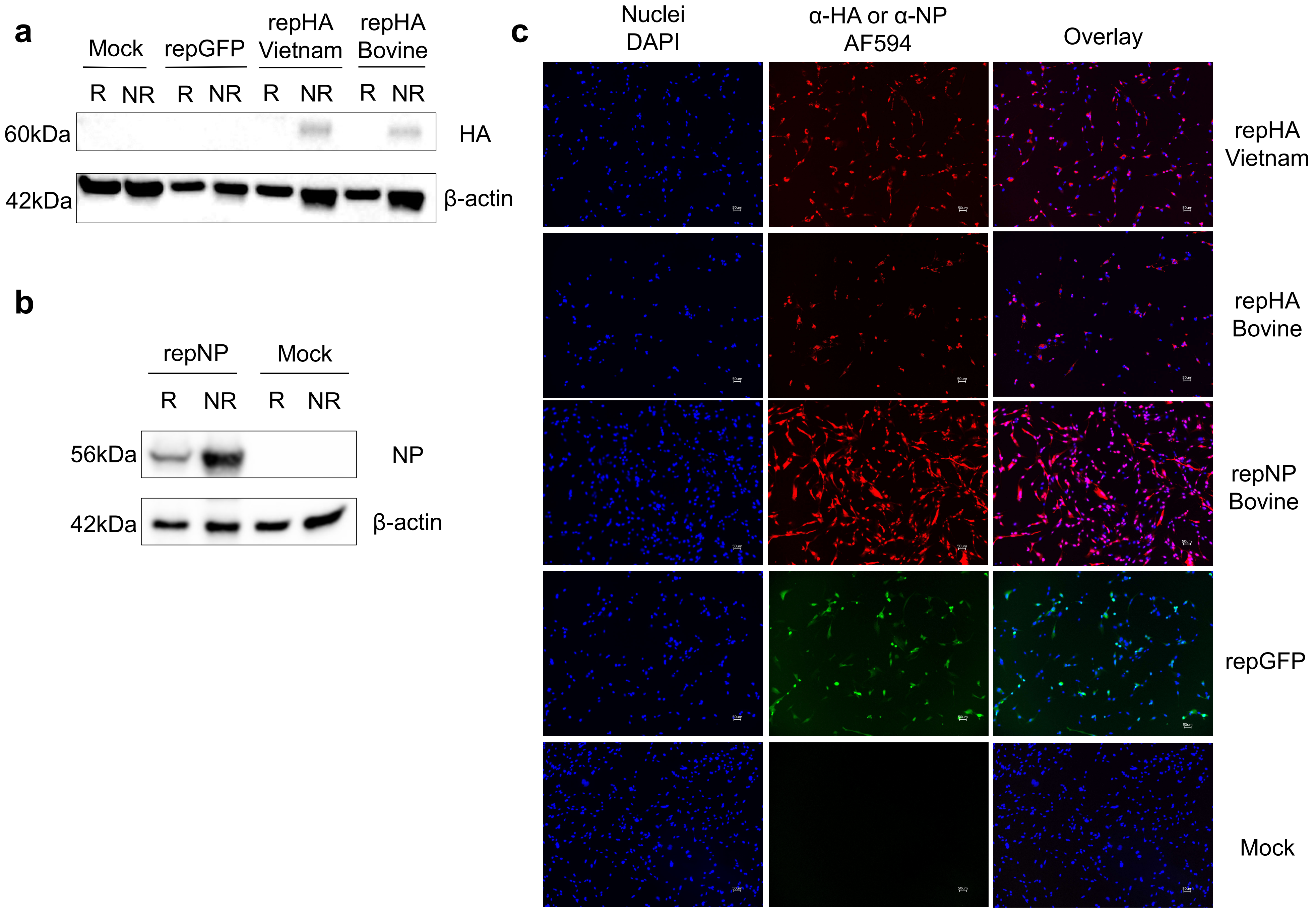

Supplemental Figure 1: RepRNA drives expression of HA and NP *in vitro.* (a-b) BHK-21 cells were transfected with the indicated repRNA and 24 hours later protein expression for HA (a) or NP (b) evaluated by western blot under reducing (R) or non-reducing (NR) conditions. Western blots performed once. (c) BHK-21 cells were transfected with indicated repRNA and 24 hours later fixed, permeabilized and stained for HA or NP. Scale bars indicate 50µm. Immunofluorescence studies performed twice

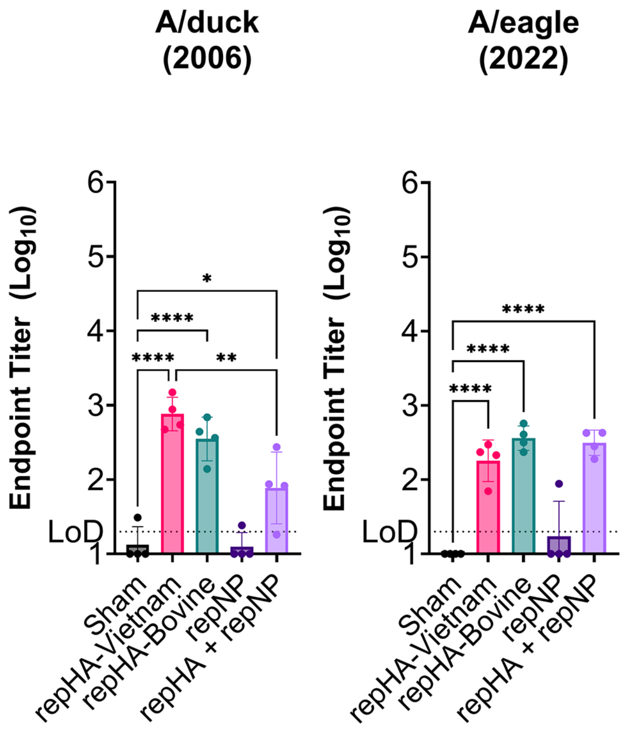

Supplemental Figure 2: repHA vaccination elicits binding antibodies to historical HAs. Binding antibodies against indicated HAs was measured by ELISA. N = 4 per group. P values calculated with a One-Way ANOVA with Tukey's multiple comparisons test. * P < 0.05, ** P <0.01, **** P < 0.0001.

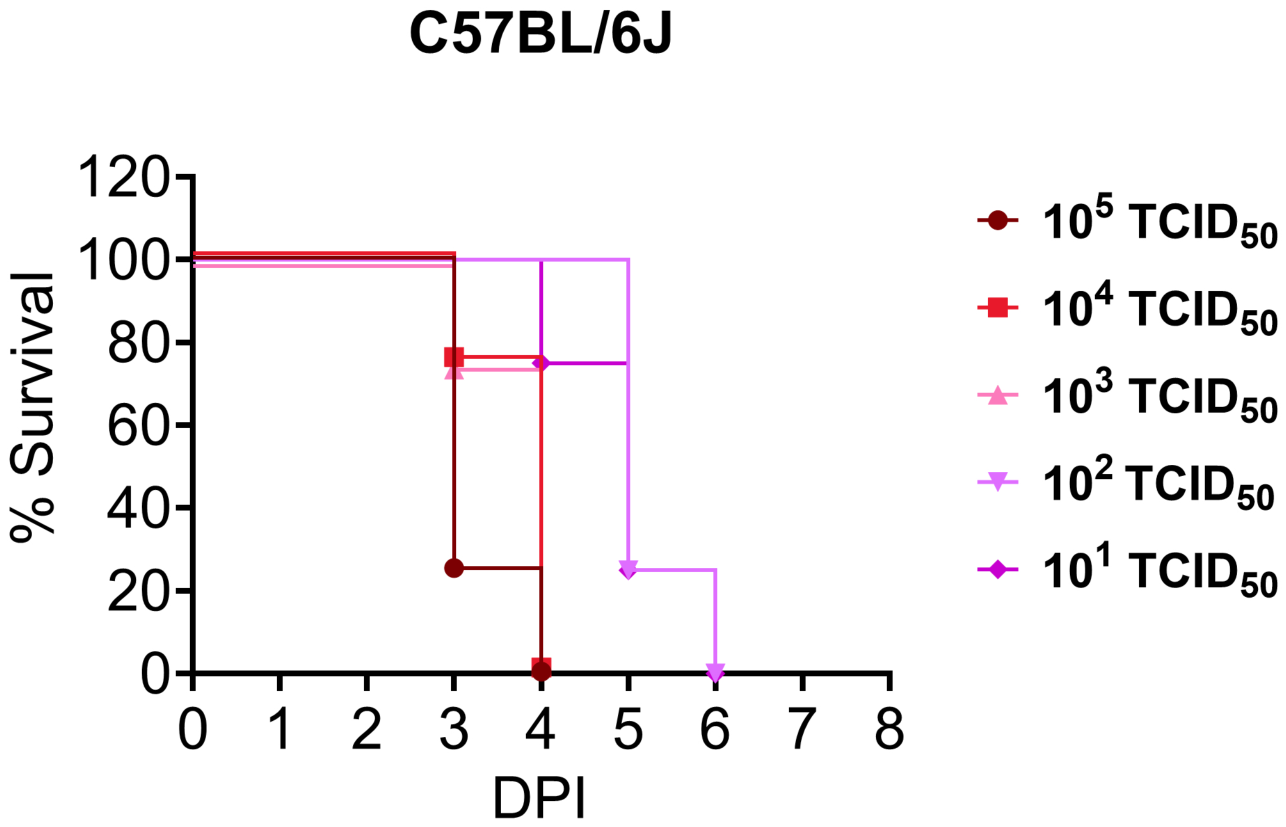

Supplemental Figure 3: C57BL6/J mice are highly susceptible to A/bovine challenge. Mice were challanged intranasally with indicated dose of A/bovine and monitored for survival. N = 4 per group.

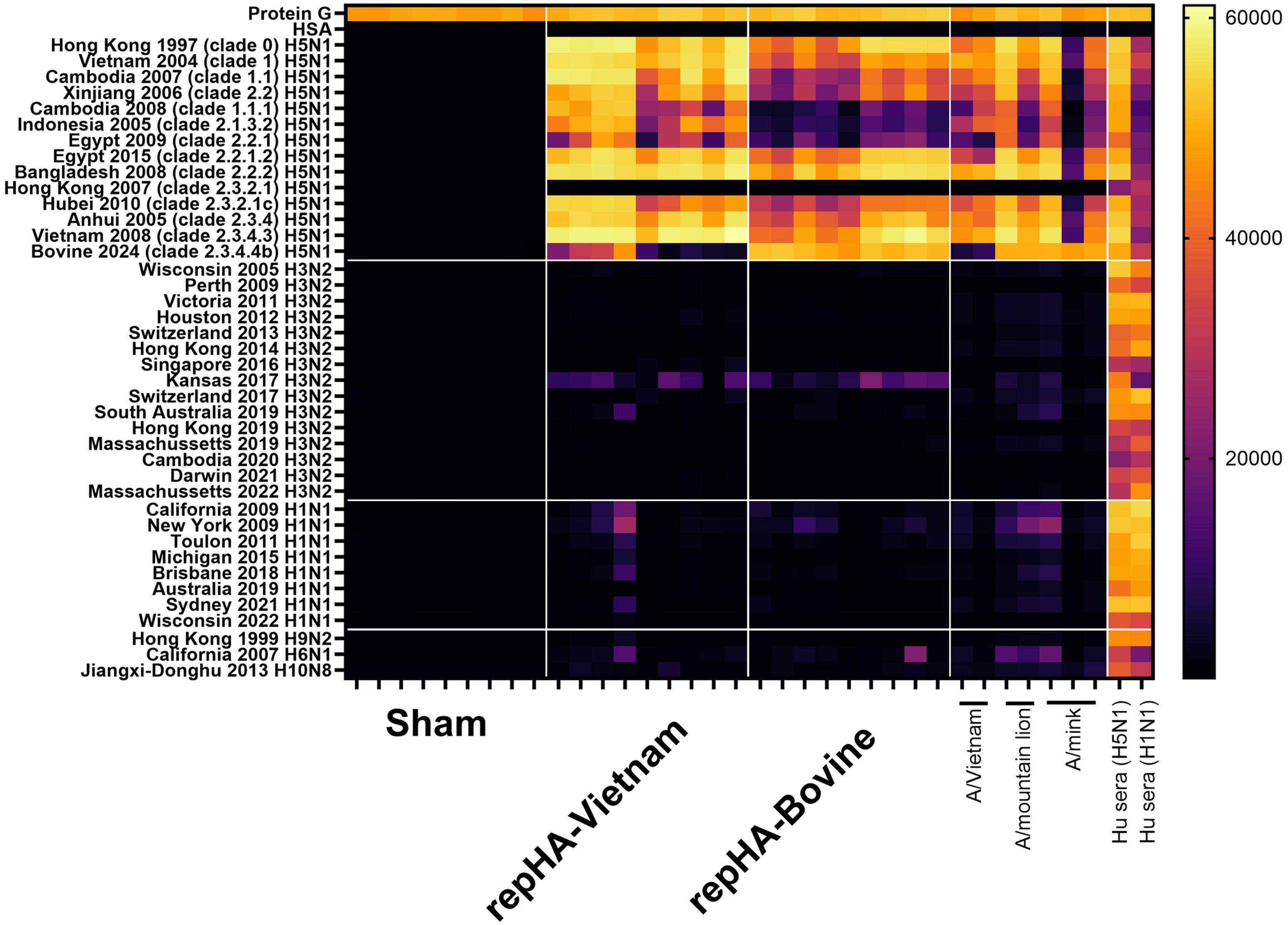

Supplemental Figure 4: Heterosubtypic immunity elicited by vaccination or infection with influenza A. Serum from vaccinated mice (sham, repHA-Vietnam or repHA-Bovine), mice that survived H5N1 challenge (A/Vietnam, A/mountain lion or A/mink), pooled from humans vaccinated with an H5N1 vaccine or from a convalescent individual infected with H1N1 in 2009 were evaluated for binding antibodies to indicated HAs in a bead-based assay. Data shown as median fluorescence intensity for individual samples. Detailed strain names are provided in supplemental table 1.

Supplemental Table 1: Antigens used in bead-based multiplex HA binding assay.

Name	Strain	Supplier	Catalog #
Egypt 2015 (clade 2.2.1.2)	A/Egypt/N0001/2015	Sino Biological	40699-V08H
Hubei 2011 (clade 2.3.2.1c)	A/Hubei/1/2010	Sino Biological	40015-V08H
Bovine 2024 (clade 2.3.4.4b)	A/dairy cow/Texas/24-008749-002	Sino Biological	41036-V08H
Anhui 2005 (clade 2.3.4)	A/Anhui/1/2005	Sino Biological	11048-V08H4
Egypt 2009 (clade 2.2.1)	A/Egypt/N05056/2009	Sino Biological	11702-V08H
Bangladesh 2008 (clade 2.2.2)	A/Bangladesh/207095/2008	Sino Biological	40697-V08H
Xinjiang 2006 (clade 2.2)	A/Xinjiang/1/2006	Sino Biological	40004-V08H
Vietnam 2008 (clade 2.3.4.3)	A/Vietnam/UT31413II/2008	Sino Biological	40022-V08H
Vietnam 2004 (clade 1)	A/Vietnam/1203/2004	IBT Bioservices	1501-001
Hong Kong 1997 (clade 0)	A/Hong Kong/483/1997	Sino Biological	11689-V08H
Cambodia 2007 (clade 1.1)	A/Cambodia/R0405050/2007	Sino Biological	11710-V08H
Indonesia 2005 (clade 2.1.3.2)	A/Indonesia/5/2005	Sino Biological	11060-V08B
Cambodia 2008 (clade 1.1.1)	A/Cambodia/S1211394/2008	Sino Biological	40026-V08H
Hong Kong 2007 (clade 2.3.2.1)	A/common magpie/Hong Kong/5052/2007	Sino Biological	40044-V08H
Perth 2009 H3N2	A/Perth/16/2009	Sino Biological	40043-V08H
Victoria 2011 H3N2	A/Victoria/361/2011	Sino Biological	40145-V08B
Texas 2012 H3N2	A/Texas/50/2012	Sino Biological	40354-V08B
Switzerland 2013 H3N2	A/Switzerland/9715293/2013	Sino Biological	40497-VNAB
Hong Kong 2014 H3N2	A/Hong Kong/4801/2014	Sino Biological	40555-V08B
Singapore 2016 H3N2	A/Singapore/INFIMH-16-0019/2016	Sino Biological	40580-V08H
Switzerland 2017 H3N2	A/Switzerland/8060/2017	Sino Biological	40949-V08B
Kansas 2017 H3N2	A/Kansas/14/2017	Sino Biological	40720-V08H
South Australia 2019 H3N2	A/South Australia/34/2019	Sino Biological	40950-V08B
Hong Kong 2019 H3N2	A/Hong Kong/45/2019	Sino Biological	40765-V08H2
Cambodia 2020 H3N2	A/Cambodia/e0826360/2020	Sino Biological	40789-V08H
Darwin 2021 H3N2	A/Darwin/6/2021	Sino Biological	40868-V08B
Massachusetts 2022 H3N2	A/Massachusetts/18/2022	Sino Biological	40992-V08H1
Wisconsin 2005 H3N2	A/Wisconsin/67/2005	Sino Biological	11972-V08H
Massachusetts 2019 H3N2	A/Massachusetts/18/2019	Sino Biological	40787-V08H
California 2009 H1N1	A/California/04/2009	Sino Biological	11055-VNAB
Michigan 2015 H1N1	A/Michigan/45/2015	Sino Biological	40567-V08H1
Brisbane 2018 H1N1	A/Brisbane/02/2018	Sino Biological	40719-V08H
Wisconsin 2019 H1N1	A/Wisconsin/588/2019	Sino Biological	40787-V08H
Sydney 2021 H1N1	A/Sydney/5/2021	Sino Biological	40944-V08B
Wisconsin 2022 H1N1	A/Wisconsin/67/2022	Sino Biological	40940-V08H7

New York 2009 H1N1	A/New York/18/2009	Sino Biological	40009-V08H
Toulon 2011 H1N1	A/Toulon/1173/2011	BEI Resources	NR-34587
Hong Kong 1999 H9N2	A/Hong Kong/1073/99	Sino Biological	11229-V08H
California 2007 H6N1	A/northern shoveler/California/HKWF115/2007	Sino Biological	11723-V08H
Jiangxi-Donghu 2013 H10N8	A/Jiangxi-Donghu/346/2013	Sino Biological	40359-V08B
