# Supplementary material for: Clade 2.3.4.4b but not historical clade 1 HA replicating RNA vaccine protects against bovine H5N1 challenge in mice
Source: Nat Commun. 2025 Jan 14;16:655. doi: 10.1038/s41467-024-55546-7 (PMC11732985; doi:10.1038/s41467-024-55546-7)
Supplement: Supplementary file 4 — Source Data [file 41467_2024_55546_MOESM4_ESM.pdf]

Figure 1a

		A/Vietnam					A/bovine		
Sham	pHA-Vietna	epHA-Bovir	repNP	repHA + repNP	Sham	repHA-Vietnam	epHA-Bovir	repNP	repHA + repNP
10	92391	1685	10	2150	50	50177.71	83268.48	50	39948.64
10	81162	529	10	2585	50	12928.48	70979.15	50	82782.2
10	63520	2435	10	1733	50	42348.75	64513.54	50	68343.71
10	98885	1254	10		50	27786.6		50	63415.4

Figure 1b

	NP	
Sham	repNP	pHA + repNP
10	17428.79	2661.371
10	5458.632	2638.944
10	8914.546	2061.713
10	11566.81	1962.972

Figure 1c

		A/Vietnam		
Sham	pHA-Vietna	epHA-Bovir	repNP	pNP + repHA
10	20	10	10	10
10	20	10	10	10

		A/bovine		
Sham	pHA-Vietna	epHA-Bovir	repNP	pNP + repHA
10	10	40	10	10
10	10	20	10	10

Figure 1d

		Sham					repHA-Vietnam					repHA-Bovine					repNP					repNP + repHA		
10	12.66667	10	10	10	107.3333	128.6667	764	1094.667	1170	857.3333	1143.333	894	709.3333	299.3333	28	23.99999	10	10	27.33333	478	288.6667	276.6667	1131.333	725.3333

Figure 1e

DPI			Sham						repHA-Vietnam						repHA-Bovine						repNP						repHA + repNP			
0	100	100	100	100	100	100	100	100	100	100	100	100	100	100	100	100	100	100	100	100	100	100	100	100	100	100	100	100	100	100
1	99.3	101.2	100.3	102.6	98.7	96.5	101.1	99.3	99	101	98.5	101	101.9	102.3	100.8	99.5	101.5	97.2	102.1	101.8	101.1	101.9	102.1	96.2	102.4	101.2	101.3	98.1	101.4	103.4
2	99.6	100.4	101.4	95.8	97.8	93.6	98.5	101.1	99	102.6	92.9	96.6	103.5	101.9	102.8	102	99.5	97.2	103.3	102.5	100.4	100.5	96.4	92.8	101	101.6	102	99.1	99.5	101.5
3	98.6	94.6	100	88.5	95.6	85.6	96.2	98.9	99.3	106.2	85.7	93.2	102.3	103.1	101.6	103.9	99.5	100.5	102.5	102.2	101.4	95.3	89.7	90	101.4	100.4	102.7	100.9	100.5	103.4
4	97.5	88.8	92.9	81.3	90.3	78.7	93.2	98.6	99.7	106.7	82.1	90.3	103.5	102.3	100.8	101.5	102.5	103.3	102.1	101.4	100.7	92	83.1	86.1	100.7	100.4	102.3	99.5	100	104.4
5	91.7		86.7		83.7		89.8	91.8	101	104.6	77.6	83.1	103.1	102.7	102	102.4	105	104.2	97.1	98.6	100.7	85.9	77.9	78.9	101.4	102.4	103	100.5	102.9	106.3
6	84.1		78.2		78.4		88.7	85.1	102.3	105.7		77.8	103.1	103.5	102	103.4	98.5	100.5	85.6	89.5	89.2	83.6		77.5	101.4	106.7	103.7	99.1	98.6	101.5
7							81.6	79.4	102.3	110.8			103.5	103.5	102.4	105.4	99.5	99.5							102.4	103.2	104	100.5	101	104.4
8									99.7	113.4			104.3	103.5	102.4	104.4	102.5	102.8							102.8	102.8	103.7	100	103.8	110.7
9									99.7	112.4			105.8	102.7	101.6	101.5	103.5	103.8							103.5	104.7	105.3	104.3	103.8	111.2
10									100.7	108.8			105.4	103.9	102.8	101	100.5	101.9							104.2	104.3	106.6	104.3	100	105.4
11									100.3	110.8			104.7	103.9	102	103.9	102	102.4							105.9	105.1	106	106.2	104.3	108.8
12									101.3	110.3			104.7	103.9	99.6	103.9	101	101.4							106.3	105.1	106.6	102.4	106.2	108.3
13									102	110.8			104.7	101.9	99.6	104.4	102	101.4							105.2	104.3	104.7	102.4	104.3	108.3
14									102	109.3			104.3	101.6	99.6	103.9	100.5	101.9							104.9	107.9	105.6	104.3	101	105.4

Figure 1d

DPI	Sham	repHA-Vietnam	epHA-Bovir	repNP	pHA + repNP
7	1				
6	1				
7	1				
5	1				
7	1				
6	1				
8		1			
8		1			
14		0			
14		0			
6		1			
7		1			
14			0		0
14			0		0
14			0		0
14			0		0
14			0		0
14			0		0
7				1	
7				1	
7				1	
7				1	
6				1	
7				1	

Figure 3a-b					Sham									repHA-Vietnam
Protein G	47073	46939.5	48978.5	49455	49242	47624	47629	49302	45814.5	49511	51522	52540.5	51336	50955
HSA	177	155	154	224	301.5	238	336	245.5	130.5	492	1285.5	652.5	645.5	217
Hong Kong 1997 (0)	233	116	135	173.5	196	161	159	182	112	58356	57554	57829	58323	46529
Vietnam 2004 (1)	302	268	239	317	316.5	290	246	264	215.5	56334	56644.5	55751	54745	49686
Cambodia 2007 (1.1)	108	74	90	124	157	142	149.5	129	88	57767	57424	56718	56902	37782
Cambodia 2008 (1.1.1)	85.5	73	89	118	246	137.5	185	188	72	51102	47832	53098	53493.5	24541
Indonesia 2005 (2.1.3.2)	118.5	90	101.5	150	197.5	183.5	226	170	95	43789	49609	52220.5	50252.5	19280
Xinjiang 2006 (2.2)	112	81.5	94	135	163	137	137.5	144	82	48335	51390.5	53859	53091	27203
Egypt 2009 (2.2.1)	157	114.5	108	160	248.5	207	282	185	103	18562.5	35589	48758	42580	6976
Egypt 2015 (2.2.1.2)	214	124	108	143	279	148.5	219	166	87	50979	54447.5	56735	55062	44731.5
Bangladesh 2008 (2.2.2)	119	75	94	99	177	115	122	110	195	56446.5	56194	57155	55487	54182
Hong Kong 2007 (2.3.2.1)	175	172	239	280	272	251	215	236	159	390	369.5	461	414	298
Hubei 2010 (2.3.2.1c)	228	118	134	187	206	171	184	185	115.5	54904	54425	55088.5	54716.5	33064
Anhui 2005 (2.3.4)	159	97	176	178	197.5	163	133	153	95	52272	55387	54421	54035	44997
Vietnam 2008 (2.3.4.3)	180.5	106.5	147	165	165	169	139	174	104.5	57757	58937	58956	58371.5	51342
Bovine 2024 (2.3.4.4b)	76.5	77	83	108	133	103	114	341.5	63	20725.5	31018	33903	47226	11551.5

Figure 3a-b									repHA-Bovine					ı Sera (H5N1)
Protein G	53872	54169.5	50947	52681.5	51167.5	52771	54164	50281	50993	51734	53248	53454	54349	52036
HSA	405	568	400	429	313	357	270	267.5	282	510	392	342.5	409.5	1424
Hong Kong 1997 (0)	51599	55891	50876	57341	44316.5	38763	47476.5	37512	48041	56063	55603.5	55563	55429	49198
Vietnam 2004 (1)	53478.5	56211	52154	57712	41221	33236	45117	35649	33826	48752.5	46497	48364	45468	50162
Cambodia 2007 (1.1)	45642.5	56960	48344	58398	29917.5	16861	29176	25428	22619	42360.5	35034	42506	34839	49326
Cambodia 2008 (1.1.1)	26009	34467	16683.5	42262.5	4294	3451	7359	10640	2660	19286	11915	17478	8850	52853
Indonesia 2005 (2.1.3.2)	30065	48561	39373	46898	6512	4657	10222.5	8447	4932	14635.5	11074	16382	8271	41025.5
Xinjiang 2006 (2.2)	47423	52393	43158	52986	25118	18593	30420.5	19082	26818	43638	36900	47189.5	36452	53802.5
Egypt 2009 (2.2.1)	30014	33193	11699	39527	11809	5622	19870	10464.5	3248	19091	20487	23613	11163	49602
Egypt 2015 (2.2.1.2)	54089	54920	50068.5	56277	41679	33917	47317.5	40255	42342	53788.5	54036	54517	53736	55334
Bangladesh 2008 (2.2.2)	56573	57036.5	54611.5	58629	49445	42912	54509	47160.5	52346	55952	56738	56406.5	55588	52400.5
Hong Kong 2007 (2.3.2.1)	330.5	719	338.5	546	385	369	384.5	387	323	418	396	375.5	364.5	54103
Hubei 2010 (2.3.2.1c)	37776.5	46548	43937.5	47958	31876	25055	35020	25628	31136	42402	42389.5	42832	43431	53063
Anhui 2005 (2.3.4)	54109	55649.5	48433	57147	36460	32327	45015	38167	33572.5	49476.5	52236	53350	44503	49027.5
Vietnam 2008 (2.3.4.3)	58620	57812	57450	61125	41443	39912	49114	42204.5	46653	55018	57529.5	59635	55384.5	48868
Bovine 2024 (2.3.4.4b)	2550	6975	5009	4080	52469.5	53310	51489	49371	46962	52261	50331	52436.5	51171.5	21859.5

	A/Vietnam		A/mountain lion			A/mink		Hu Sera (H1N1)
Protein G	46135	48839	52541.5	50255	52432.5	46580	48117	51579
HSA	1535	946	2868	2652	3853	881	2109	1509.5
Hong Kong 1997 (0)	40878	45368	56298	48309	55241	11204	41797	26494
Vietnam 2004 (1)	49684	47408	53957	45865	52650	15134.5	42468	33096
Cambodia 2007 (1.1)	39134	44597	53083	33743.5	51558	4709.5	31056	25281
Cambodia 2008 (1.1.1)	12857	32495	39694	15955	39394	1482	18234	12517.5
Indonesia 2005 (2.1.3.2)	28413	39252	40940	10713	33208.5	1909	20034.5	15799
Xinjiang 2006 (2.2)	30556.5	35034	51445	27910	44725	5530	28298	19200
Egypt 2009 (2.2.1)	14519	7225.5	40608.5	15300	38045.5	3550	23999	19768
Egypt 2015 (2.2.1.2)	34003	26059	55340	46195	53721	11470	41125	18755
Bangladesh 2008 (2.2.2)	48413	50694	56557.5	53228	55658	14379	45827	23877
Hong Kong 2007 (2.3.2.1)	1322	557	1670	2020	2514.5	443	1103	29268
Hubei 2010 (2.3.2.1c)	35755	42086	51690.5	38743	50923	6925	32773	26396
Anhui 2005 (2.3.4)	45093	41233	54083	47991	52747	15024	42984.5	27736
Vietnam 2008 (2.3.4.3)	46610.5	49703.5	57734	50289	56831.5	12843	45398.5	21654
Bovine 2024 (2.3.4.4b)	5944	9440	50077.5	50087	50339	47894	49481.5	31260
